# Clinical significance of left atrial volume in clinical outcomes of heart transplant recipients

**DOI:** 10.1186/s13019-015-0308-8

**Published:** 2015-07-11

**Authors:** Saad Ahmad, Pradeep Gujja, Tehmina Naz, Jun Ying, Stephanie H. Dunlap, Yukitaka Shizukuda

**Affiliations:** 1 Division of Cardiovascular Disease and Health, Department of Internal Medicine, University of Cincinnati, 231 Albert Sabin Way, ML0542, Cincinnati, OH 45267 USA; 2 Department of Public Health Sciences, University of Cincinnati, 160 Panzeca Way, Cincinnati, OH 45267 USA; 3 Division of Cardiology, Cincinnati VA Medical Center, 3200 Vine Street, Cincinnati, OH 45220 USA

**Keywords:** Left atrial volume, Heart transplant, Clinical outcomes, Echocardiography, Retrospective study

## Abstract

**Background:**

Left atrial volume (LAV) is surgically kept enlarged in heart transplant (HT) recipients. On the other hand, LAV has been known an independent predictor of various cardiovascular diseases and is associated with exercise capacity of HT recipients. Thus, we evaluated the hypothesis that LAV is still associated with clinical outcomes in HT recipients whose left atria are artificially enlarged.

**Methods:**

Clinical outcomes over 5 years after HT were retrospectively evaluated in 35 HT recipients who had a LAV measurement with echocardiography at 1 year after HT at the University of Cincinnati Medical Center. The LAV was derived from a stacked disc method using apical 4 and 2 chamber views.

**Results:**

The average LAV normalized to body surface area was 38.3 ± 9.9 ml/m^2^ (mean ± SD) at 1 year after HT. Two deaths and one drop-out occurred during 5-year follow up. A total of 552 cardiac symptom-related hospitalizations occurred in the recipients. The average time to first hospitalization was 166 ± 279 days and average number of hospitalizations of each recipient was 15 ± 16. The indexed LAV failed to correlate with the time to first hospitalization and number of hospitalizations of each recipient (Spearman’s *p*-value; 0.141 and 0.519 respectively). When the patients were divided to groups of large LAV (*n* = 17) and small LAV (*n* = 18) using the cut off value of the mean LAV, no significant difference was noted in mortality, hospitalization, and new onset of atrial fibrillation between the groups.

**Conclusions:**

Although our study is limited by a retrospective study design and relatively small number of patients, our results implicate that LAV is not significantly associated with clinical outcomes in HT recipients over 5 years after HT.

## Background

Left atrial volume (LAV) is enlarged in heart transplant (HT) recipients [1, 2]. Enlarged LAV seems negatively correlated with exercise capacity [1] and it may be detrimental to clinical outcomes in HT recipients since limited exercise capacity has known to be a poor prognostic factor in various cardiovascular diseases such as heart failure and atrial fibrillation [3–5]. In addition, it is unknown whether LAV can still correlate with clinical outcomes in the setting of artificially enlarged LAV because previous published studies examining a predictive value of LAV in clinical outcomes only have been described in altered LAV naturally by disease processes [6–8]. Therefore, a clinical relevance of artificially dilated LAV to clinical outcomes is largely unknown.

In this study, we tested whether artificially enlarged LAV by HT is still associated with clinical outcomes in this population to address the above concerns.

## Methods

### Study population

This clinical protocol was approved by the Institutional Review Board and was consistent with the principles of the Declaration of Helsinki [9]. Due to the retrospective nature of the study, waiver of consent was approved. Patients with heart failure who underwent post HT clinical follow up were included when the following conditions were met: 1) Post HT follow up was performed in our institution, 2) Baseline post-HT echocardiography was performed at 12 months of post HT, 3) No more than mild mitral regurgitation during baseline echocardiography, 4) No clinically significant myocardial ischemia with stress testing at the time of study entry, 5) Normal sinus rhythm, 6) No clinically significant active transplant rejection at the time of study entry. LAV was measured at 12 months from HT to avoid the effect of acute hemodynamic changes due to the HT surgical procedure. Thirty-five patients who visited our clinic for a post HT follow up between 2000 and 2007 met the inclusion criteria. All cases received standard left atrial cuff anastomosis.

### Echocardiographic measurements

The patients were imaged with multi-frequency transducers with center frequencies of 2.5 or 3.5 MHz (iE33, Philips Medical System, Bothell WA, Vivid 7 GE Healthcare system, Milwaukee, WI). Studies were recorded digitally and stored in the Camtronics Imaging system (Emageon Camtronics system, Birmingham AL). LAV measurements were performed off-line on digital loops using a Digisonics review station (version 3.2 software, Digisonics Inc. Houston, TX) as previously reported by our group [1, 7, 10, 11]. LAV were measured using apical 4 and 2 chamber views at end systole using a stacked disc method. Briefly, in all cases pulmonary veins and the LA appendage were excluded from planimetric analysis. The outline of the atrial endocardium was traced at the end of ventricular systole at the point of maximum left atrial dimension. We used this method over the area-length method recommended by the American Society of Echocardiography [12] to calculate LAV because our method is based by fewer geometric assumption than the area-length method. The studies were blinded and measured by a single reader (Y.S.).

### Clinical outcomes

Over 5-year period from HT, the medical chart of each patient was retrospectively reviewed. Frequency of death, cardiac symptom-related hospitalization, and atrial fibrillation were recorded for the analyses. The cardiac symptom-related hospitalization was defined as a hospitalization due to shortness of breath, chest pain, dizziness, palpitations, and peripheral edema which was accompanied with an admitting physician’s note indicating a suspected cardiac cause. The time to first admission was measured as previously described [13, 14].

### Statistical analysis

Data are presented mean ± S.D. for measurements. The relationship between LAV and clinical outcome variables were analyzed by a Spearman correlation test. Event free and survival analyses between the groups were made by a Kaplan-Meyer analysis. Clinical outcomes between the groups were compared with a χ-square test. All tests were two-sided and analyses with a *p*-value <0.05 were considered statistically significant.

## Results

### Patients’ characteristics

The average age of study population at the time of HT was 52 ± 16 years. Ten were female (29 %). Among the patients investigated, most were asymptomatic. Twenty-eight patients (80 %) were NYHA class I and no patient showed clinically significant rejection (International Society for Heart and Lung Transplantation grade II or more). Baseline echocardiography showed that the average LAV was 77.3 ± 21.6 ml and LAV normalized to body surface area (BSA) was 38.3 ± 9.9 ml/m^2^. Both values were higher than our historical normal values [11], reflecting typical HT morphology. One mildly to moderately reduced and two mildly reduced left ventricular systolic function were reported in this cohort. One mild and two moderate left ventricular diastolic dysfunction were reported in this cohort; however, left ventricular diastolic function was not reportable in 6 patients. Seventeen patients (49 %) demonstrated indexed LAV more than the average value of this group.

### Clinical outcomes

A total of 552 cardiac symptom-related hospitalizations occurred in this group. No hospitalization was noted in 4 patients. The average time to first hospitalization was 166 ± 279 days and average number of hospitalizations of each recipient was 15 ± 16. Two deaths and 2 post HT atrial fibrillation were noted over 5-year retrospective clinical follow up in this group.

### Relationship between LAV and clinical outcomes

**Fig. 1:**
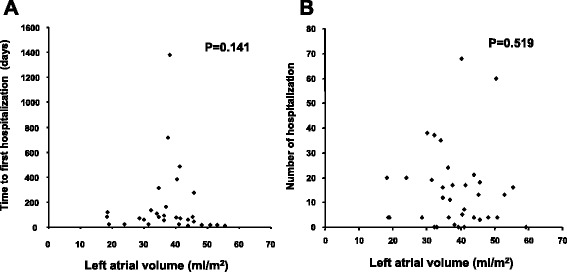
The relationships of left atrial volume normalized to body surface area with time to first cardiac-symptom related hospitalization (panel **a**, *n* = 31) and number of hospitalizations (panel **b**, *n* = 35) are shown. Spearman’s correlation *p* values are shown

**Fig. 2:**
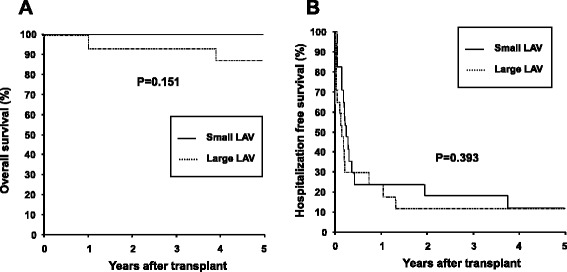
The Kaplan-Myer plots of mortality reflected by overall survival(panel **a**) and time to first cardiac-symptom related hospitalization reflected by hospitalization free survival(panel **b**) are shown. The unbroken lines denote patients with indexed left atrial volume to body surface area smaller than or equal to its average of this study population (*n* = 18). The dotted lines denote patients with indexed left atrial volume to body surface area larger than the average of this study population (*n* = 17). Please refer to the text for details. *p* values from a log-rank test are shown

The indexed LAV failed to correlate with the time to first hospitalization and number of hospitalizations of each recipient (Spearman’s *p*-value; 0.141 and 0.519 respectively; Fig. 1). The absolute LAV also did not correlate with them significantly (Spearman’s *p*-value; 0.438 and 0.560 respectively). To assess a clinical significance of LAV in clinical outcomes, the cohort was divided to patients with LAV > the average value (38.3 ml/m^2^, LL group, *n* = 17) and patients with LAV ≤ the average value (SL group, *n* = 18). Two deaths, 256 hospitalization, and 1 post transplant atrial fibrillation occurred in LL group. No death, 266 hospitalization, and 1 post transplant atrial fibrillation occurred in SL group. These incidences were statistically not different between the groups. Both mortality reflected by overall survivaland time to first cardiac symptom-related hospitalization reflected by hospitalization free survivaldid not defer statistically between LL and SL groups with a Kaplan-Meier analysis (log-rank test; 0.151 and 0.393 respectively; Fig. 2).

## Discussion

The results of the present study demonstrate that in this cohort of HT patients, the size of left atrium measured with LAV is not associated with clinical outcomes measured with mortality, hospitalization, and atrial fibrillation over 5-year period after HT.

Although larger LAV is associated with lesser exercise capacity in our previous publication [1], LAV fails to associate clinical outcomes in this study. LAV has been previously reported to correlate with clinical outcomes in variety of cardiac pathologies including coronary artery disease [15, 16] and heart failure [8, 17]. But this relationship has not been tested in the setting of surgically-altered, artificially-dilated left atrium and our results show that this relationship is not established although our patient population is small and may not identify weak associations. It is widely believed that dilation of LAV is a reflection of chronic degree of left ventricular diastolic function [18] and in turn chronicity of left ventricular diastolic dysfunction correlates with clinical outcomes. Transplant hearts have been reported to have normal left ventricular diastolic function irrespective of enlarged LA [1, 19] in the absence of clinically significant allograft rejection and this dissociation of left atrial size from left ventricular diastolic function at baseline may be a culprit of its failure to associate with clinical outcomes.

A decrease in exercise capacity has been known for a poor prognostic marker in heart failure [3, 5] and coronary artery disease [20]. Our results suggest that this relationship may not be applicable to HT population within 5 years from HT although LAV is associated with exercise capacity in this population like in the other cardiac diseases [1]. However, our results do not preclude the possibility that exercise capacity still affects the clinical outcomes in HT population because the other surrogate markers of exercise capacity rather than LAV were not evaluated on this study and further investigation is warranted to address this. We speculate that in HT population, conventional clinical outcome predictors may not be usable and that unknown or lesser known clinical outcome predictors may exist. For instance, factors unique to HT such as response to immunosuppressive therapy may be more influential to clinical outcomes than traditional predictors such as LAV and exercise capacity and further investigation is warranted to identify these.

However, a caution should apply to our observation. The retrospective nature of this study and relatively small sample size are the primary limitations of the present investigation. The 5-year observation period may also not be sufficient to conclude an accurate assessment of predictive value of LAV in clinical outcomes in our HT recipients. Although not statistically significant, a small number of deaths were only noted in the patients with large LAV group, not those with small LAV. This difference between the groups may reach a statistical significance if this analysis is repeated after longer duration of observation time than this study. To further strengthen our findings, a prospective study addressing these issues in a larger HT cohort over longer time period is required.

## Conclusion

In conclusion, our study shows that LAV is not significantly associated with clinical outcomes in HT recipients over 5-year follow up from HT. Further investigation to verify this observation is therefore warranted.

## Abbreviations

LAV: Left atrium volume
HT: Heart transplant
BSA: Body surface area
